# KDM5C-regulated SIX5 promotes glioblastoma progression through transcriptional activation of UBE2C and enhancement of the Warburg effect

**DOI:** 10.3389/fimmu.2026.1788510

**Published:** 2026-03-20

**Authors:** Zhang Li, Nan Wang, Defeng Liu, Yunshan Wang, Haiying Li

**Affiliations:** Medical Research and Laboratory Diagnostic Center, Central Hospital Affiliated to Shandong First Medical University, Jinan, China

**Keywords:** AKT/mTORsignaling, glioblastoma, KDM5C, SIX5, therapeutic target, transcriptional regulation, UBE2C, Warburg effect

## Abstract

Gliomas are the most common primary malignant tumors of the adult central nervous system, characterized by rapid growth, high recurrence rates, and limited response to standard treatments, with median survival under 15 months. The SIX transcription factor family has been implicated in tumor development, but the role and regulatory mechanism of SIX5 in glioblastoma (GBM) remain unclear. This study systematically investigates the biological function of SIX5 and its regulatory network in GBM. Differential expression and weighted gene co-expression network analyses of GSE4290 and GSE50161 datasets, combined with machine learning algorithms including LASSO, identified SIX5 as a core candidate gene. Functional enrichment analyses and evaluation using TCGA and UALCAN databases revealed that SIX5 is highly expressed in GBM and associated with poor prognosis. Single-cell RNA sequencing and spatial transcriptomics showed enrichment of SIX5 in the tumor core and in astrocyte-like and stem cell-like subsets at the invasion front. *In vitro*, U87 and U251 cells with lentivirus-mediated SIX5 knockdown or overexpression were assessed for proliferation, migration, invasion, apoptosis, and colony formation. SIX5 knockdown significantly inhibited proliferation, migration, invasion, epithelial-mesenchymal transition, and tumorigenicity, while promoting apoptosis. Mechanistically, KDM5C positively regulates SIX5, which directly binds the UBE2C promoter to activate its transcription, enhancing AKT/mTOR signaling and promoting aerobic glycolysis via upregulation of GLUT1, HK2, PGK1, and LDHA. Rescue experiments showed that UBE2C overexpression partially restored malignant phenotypes under SIX5 downregulation. *In vivo* xenograft studies confirmed that the KDM5C–SIX5–UBE2C axis drives GBM growth. In conclusion, SIX5 functions as a critical oncogenic driver in GBM, regulated by KDM5C and promoting tumor progression through UBE2C-mediated activation of AKT/mTOR signaling and glycolytic reprogramming. The KDM5C–SIX5–UBE2C regulatory axis represents a potential prognostic biomarker and therapeutic target in glioblastoma.

## 1 Introduction

Gliomas are the most common primary tumors of the central nervous system (CNS) in adults, accounting for more than 70% of all malignant brain tumors (1). Clinically, gliomas are mainly classified into oligodendrogliomas, astrocytomas, and glioblastomas (2). According to the fifth edition of the classification criteria for tumors of the central nervous system published by the World Health Organization (WHO) in 2021, adult diffuse gliomas are mainly divided into the following three types: astrocytoma (IDH-mutant type, WHO grade 2–4), oligodendroglioma (IDH-mutant type with 1p/19q combined deletion, WHO grade 2–3) and glioblastoma (IDH wild type, WHO grade 4) (3). GBM typically exhibits highly invasive growth within the brain parenchyma (4), subsequently infiltrating blood vessels and nerves, which facilitates its dissemination within the CNS (5). Current standard treatment strategies include surgical resection, radiotherapy, and adjuvant chemotherapy with temozolomide (TMZ) (6). Low-grade gliomas (grades I–II) generally display slower growth, lower invasiveness, better prognosis, and higher sensitivity to treatment (7). In contrast, GBM is characterized by pronounced aggressiveness and lethality, frequent recurrence, and resistance to both radiotherapy and chemotherapy, leading to poor therapeutic outcomes (8). Globally, both the incidence and mortality of GBM remain high and show a rising trend (9). The overall prognosis of patients is dismal, with a median survival time of less than 15 months following diagnosis (10). Therefore, elucidating the molecular mechanisms underlying GBM and identifying novel therapeutic targets are of great significance for improving patient outcomes and developing precision therapies.

The SIX gene family is a highly conserved class of development-related transcriptional regulators that play a crucial role in organogenesis, cell differentiation, and various pathological processes (11). This family mainly includes SIX1, SIX2, SIX3, SIX4, SIX5, and SIX6, whose encoded proteins can positively or negatively regulate the transcription of downstream target genes, finely modulating the expression program of development-related genes (12, 13). Previous studies have shown that members of the SIX family exhibit aberrant expression in various tumors and are closely related to chromatin binding capacity and DNA-dependent transcriptional regulation activity. For example, in colorectal cancer, aberrant activation of SIX proteins is considered closely related to tumorigenesis and development (14). Recent studies have further revealed that the SIX family is generally imbalanced in expression in various malignant tumors such as melanoma, ovarian cancer, glioma, hepatocellular carcinoma, and lung cancer (15–19). It is noteworthy that, as a typical developmental regulator, SIX1 can significantly enhance the proliferation and invasion of glioblastoma cells by transcribedly activating connective tissue growth factor (CTGF) (20), suggesting that members of the SIX family may have important biological significance in GBM progression. However, the specific function of SIX5 in GBM and its potential molecular regulatory mechanism still lack systematic research. Based on the results of bioinformatics analysis, this study speculates that SIX5 may be involved in regulating the transcriptional activity of the cell cycle-related gene UBE2C. In view of this, this study aims to comprehensively analyze the biological role of SIX5 in glioblastoma and further elucidate its upstream and downstream regulatory network and potential tumorigenic mechanism.

This study systematically demonstrated the pro-tumorigenic role of SIX5 in glioblastoma. Compared with normal brain tissue, SIX5 was significantly upregulated in GBM, and its elevated expression level was closely associated with poor clinical outcomes in patients. Further functional and mechanistic studies showed that SIX5 expression is regulated by the upstream epigenetic regulator KDM5C, and SIX5 promotes the formation and maintenance of malignant phenotypes in tumor cells by directly activating the transcription of the cell cycle-related gene UBE2C. This process is accompanied by the sustained activation of the AKT/mTOR signaling pathway and the remodeling of tumor metabolism towards aerobic glycolysis. *In vivo* and *in vitro* experimental results consistently showed that the KDM5C – SIX5 – UBE2C regulatory axis plays a key driving role in the growth and progression of glioblastoma. In conclusion, SIX5 may serve as a prognostic biomarker with potential clinical value and provide new molecular evidence for targeted therapy of GBM.

## 2 Materials and methods

### 2.1 Data acquisition and differential expression analysis

This study obtained glioma-related gene expression profiles from the Gene Expression Omnibus (GEO) database, including two datasets: GSE4290 and GSE50161. GSE4290 included 157 glioma samples (26 astrocytomas, 50 oligodendrogliomas, and 81 glioblastomas) and 23 normal brain tissue samples; GSE50161 included 34 glioblastoma samples and 13 normal brain tissue controls. Based on the R language environment, the limma software package was used for preprocessing and differential expression analysis of the above data, including background correction and data normalization. The screening threshold for differentially expressed genes was set to |log2 fold change| > 1, and the p-value after Benjamini–Hochberg correction was < 0.05. The analysis results were visualized using volcano plots and heatmaps.

### 2.2 Weighted gene co-expression network construction and machine learning-based feature selection

A gene co-expression network was constructed using the WGCNA software package. First, all genes were initially screened based on median absolute deviation (MAD), retaining only the top 25% of genes with the highest degree of variation for subsequent analysis. Then, an appropriate soft threshold power parameter was selected to ensure the network conformed to the characteristics of a scale-free topology. Co-expression modules were identified using dynamic tree pruning, and the minimum number of genes in each module was set to 30. Next, the Pearson correlation coefficients between the characteristic genes of each module and the glioma phenotype were calculated, and modules with an absolute correlation coefficient greater than 0.5 and a p-value < 0.01 were defined as core modules significantly associated with the disease.

Based on this, differentially expressed genes from the two datasets were integrated with the genes from the aforementioned core modules using a Venn diagram to obtain a preliminary candidate gene set. Further, multiple machine learning algorithms were employed to screen candidate genes for features, including: LASSO regression analysis (glmnet package, using 10-fold cross-validation), random forest model (randomForest package, ntree = 500), support vector machine recursive feature elimination algorithm (SVM-RFE, e1071 package, radial basis function kernel), and extreme gradient boosting model (XGBoost, max_depth = 6, eta = 0.3, nrounds = 100). Finally, the core predicted gene was determined by taking the intersection of the screening results of the above four algorithms through Venn diagram. The results showed that SIX5 had high importance in all models and was identified as a key candidate gene.

### 2.3 Functional enrichment and pathway analysis

All gene functional annotations and pathway enrichment analyses were performed using the R language environment. For target genes, Gene Ontology (GO) and KEGG pathway enrichment analyses were performed using the clusterProfiler package. GO annotations covered three levels: biological processes, cellular components, and molecular functions. Enrichment significance was tested using a hypergeometric distribution model, and multiple comparison correction was employed to control for false positives. Results showed that enrichment was considered statistically significant when the corrected P-value was < 0.05 and the false discovery rate (FDR) was < 0.05. The analysis results are presented in bubble charts and bar charts. Unless otherwise specified, all statistical calculations were performed using R language, and a two-sided P-value < 0.05 was used to determine significance.

### 2.4 Bioinformatics analysis and multidimensional transcriptome data integration analysis based on the UALCAN database

Based on the UALCAN online analysis platform, a systematic evaluation of the expression characteristics and clinical relevance of SIX5 in glioblastoma was conducted. First, the expression levels of SIX5 in GBM tumor tissue and normal brain tissue were compared. Then, patients were grouped according to the median SIX5 expression value, and the Kaplan-Meier method was used to analyze the relationship between different expression levels and overall survival (OS). Furthermore, the correlation between SIX5 expression and patient clinicopathological parameters (including age and sex) was further analyzed. To further analyze the expression characteristics of SIX5 in the GBM tumor microenvironment at both single-cell and spatial dimensions, the single-cell RNA sequencing dataset GSE182109 and the spatial transcriptome dataset GSE237183 from the GEO database were integrated. Single-cell data underwent quality control, normalization, PCA dimensionality reduction, UMAP visualization, and cell subpopulation annotation using Seurat (v4.0) to clarify the expression patterns of SIX5 in different cell populations. Spatial transcriptome data were integrated using Seurat and STUtility to map SIX5 expression to tissue spatial coordinates, revealing its spatial distribution characteristics in different regions.

### 2.5 Cell lines and cell culture

Human glioblastoma cell lines U87 and U251 were used as *in vitro* experimental models in this study. All cells were purchased from the Shanghai Cell Bank of the Chinese Academy of Sciences and were confirmed to be free of contamination by routine mycoplasma testing. Human embryonic kidney HEK293T cells were also used; this adherent, epithelial-like cell line was mainly used for lentivirus packaging, viral titer determination, and related transfection experiments. All cells were maintained under standard culture conditions (37 °C, 5% CO_2_) in Durbeco modified Eagle medium (DMEM, Gibco, Life Technologies, USA), supplemented with 10% fetal bovine serum, 100 U/mL penicillin, and 100 μg/mL streptomycin.

### 2.6 Lentiviral shRNA vector construction and cell infection

Based on the coding sequences of SIX5, KDM5C, and UBE2C, this study designed and synthesized specific interference fragments targeting each gene, and subcloned them into the lentiviral vector pLKO.1 (Shanghai Bio Sci Res). Simultaneously, the full-length sequences of these three genes were inserted into the same lentiviral backbone to construct overexpression vectors. The packaged lentiviral particles were transfected into U87 and U251 glioma cells at 10 MOI using Lipofectamine^®^ 2000 (Invitrogen, Thermo Fisher Scientific, California, USA). Forty-eight hours after transfection, stable cell lines were established by selection with puromycin (2 μg/mL, Sigma-Aldrich, USA); subsequently, maintenance culture was performed at approximately 1/4–1/2 of the initial dose to promote the continuous amplification of drug-resistant clones. After the establishment of stable cell lines, total RNA was extracted, and changes in gene expression were verified by real-time quantitative PCR (RT-qPCR).

### 2.7 RNA isolation and qPCR

After collection of U87 and U251 cells, total RNA was extracted using TRIzol reagent (Invitrogen, USA). RNA concentration and purity were measured using a NanoDrop 2000/2000C spectrophotometer (Thermo Fisher Scientific, USA). RNA samples meeting quality requirements were then used as templates for reverse transcription using an M-MLV reverse transcriptase kit (Promega, USA) to synthesize complementary DNA (cDNA). Real-time quantitative PCR was then performed using SYBR Green PCR premixed reagent (Thermo Fisher Scientific, USA). Gene expression levels were relatively quantified using the 2^-^ΔΔCq method. Primer sequences are listed in **Supplementary Table 1**, and GAPDH was used as an internal reference gene for calibration.

### 2.8 Western blotting (WB) analysis

After cell collection, RIPA lysis buffer (P0013B, Beyotime, Shanghai) containing 1% PMSF was added, and cells were lysed on ice for 30 minutes. The lysis buffer was centrifuged at 14000 g at 4 °C, and the supernatant was collected. Protein concentration was determined using a BCA protein quantification kit (P0012S, Beyotime, Shanghai). Then, an appropriate amount of 5× loading buffer was added, and the protein was denatured by boiling at 100 °C for 10 minutes. 50 μg of protein was loaded into each well. After separation by SDS-PAGE, the protein was transferred to a PVDF membrane. The membrane was blocked in 5% skim milk at room temperature for 1 hour and incubated at 4 °C with primary antibody (antibody information is shown in Appendix 2), using GAPDH as an internal control. After adding uniformly distributed ECL reaction solution (AR1172, Boster Biological, Wuhan), the membrane was developed using an Amersham Imager 600 (USA). The grayscale values of the bands were quantitatively analyzed using ImageJ software. All experiments were repeated at least three times.

### 2.9 CCK-8 assay for detection of glioblastoma cell proliferation

Glioblastoma cells in the logarithmic growth phase, after different transfection treatments, were seeded at a density of 8 × 10³ cells/well in 96-well plates and cultured for 24, 48, and 72 h, respectively. At each time point, 10 μL of CCK-8 reagent (catalog number 96992, Sigma-Aldrich, USA) was added to each well, followed by incubation at 37 °C in a humidified environment for 1 h. The absorbance was measured at 450 nm using an Epoch microplate reader (Bio-Tek, USA). Each experiment was performed in 6 replicates and independently in 3 duplicates to ensure the stability and reproducibility of the results.

### 2.10 Colony formation assay

Single-cell suspensions were seeded at a density of approximately 500 cells/well in 6-well plates and cultured for 10–14 days until visible clones formed. Cells were then fixed with 4% paraformaldehyde and stained with 0. 1% crystal violet for observation. Independent cell clusters containing ≥50 cells were defined as valid colonies and manually counted under an optical microscope. Each experiment was independently repeated three times to ensure reproducibility and statistical reliability of the results.

### 2.11 Wound healing assay

U87 and U251 cells (5 × 105) were seeded into 6-well plates and cultured to full confluence. Linear scratches were introduced using a 200 µL pipette tip, followed by two washes with PBS to remove debris. Cells were then maintained in serum-free medium. Wound closure was documented at 0 h and 24 h, and the migration area was quantified using ImageJ software.

### 2.12 Transwell migration and invasion assays

For the migration assay, transwell chambers were placed in 24-well plates containing 600 μL RPMI-1640 supplemented with 10% FBS in the lower compartment. A total of 5 × 104 cells suspended in 100 μL serum-free medium were seeded into the upper chamber. After 24 h, migrated cells on the lower surface were fixed with 4% formaldehyde and stained with 0. 1% crystal violet. For the invasion assay, the upper chambers were precoated with Matrigel (Corning, USA) prior to seeding the same number of cells.

### 2.13 TUNEL staining for detection of cell apoptosis

Cells from each group were collected and seeded onto coverslips for adherent culture. After fixation with 4% paraformaldehyde for 30 minutes, cells were washed with PBS. Cells were then treated with Triton X-100 for 5 minutes to enhance membrane permeability, followed by rinsing with PBS. Cells were incubated with TUNEL reaction solution at 37 °C in the dark for 1 hour. After incubation, cells were washed again with PBS, and the nuclei were counterstained with Hoechst stain for 15 minutes at room temperature in the dark. Finally, after rinsing with PBS, coverslips were mounted, and apoptosis images were observed and acquired under a fluorescence microscope.

### 2.14 Xenograft mouse tumor model

All animal experiments in this study were conducted in strict accordance with the National Institutes of Health’s Guidelines for the Management and Use of Laboratory Animals (NIH Publication No. 85-23, 1996 revised edition) and were approved by the Ethics Committee of the Affiliated Central Hospital of Shandong First Medical University. Twenty-four 4-week-old female BALB/c nude mice (Shanghai Lingchang Biotechnology Co., Ltd.) were randomly assigned to four groups: the shCtrl group (negative control, n = 6), the shSIX5 group (SIX5 knockdown, n = 6), the OE-NC group (negative control, n = 6), and the shSIX5 + OE-UBE2C group (combined intervention, n = 6). U87 cells were prepared into a cell suspension with a concentration of 4 × 10^6^ cells/mL, and 200 μL was subcutaneously injected into the right forelimb of each mouse. From cell inoculation, tumor volume was measured every 2 days, and mouse weight was recorded simultaneously, with observation continuing until day 26. At the end of the experiment, all animals were euthanized, and the tumor tissue was completely removed for weighing and image acquisition and recording.

### 2.15 Chromatin immunoprecipitation (ChIP)

Cells were first cross-linked with 1% formaldehyde, and the reaction was terminated with 1.25 M glycine. Cells were then lysed and chromatin was obtained by sonication. The obtained chromatin was incubated overnight at 4 °C with anti-SIX5 antibody (Cell Signaling Technology, USA) or negative control normal rabbit IgG. The immune complex was then captured by ChIP-grade protein A/G magnetic beads for 2 hours, and the target protein-chromatin complex was eluted after washing. The DNA was reverse cross-linked and purified before being used for RT-qPCR analysis. Information on the antibodies and primers used is listed in **Supplementary Tables 3**, **S4**, respectively.

### 2.16 Dual - luciferase reporter assay

The binding sites of miRNAs were predicted using TargetScan (http://www.targetscan.org/). Corresponding 3’‐UTR sequences were synthesized by Genechem and cloned into reporter vectors. 293T cells were seeded in 96-well plates and transfected with either the reporter plasmid or miR-30c-5p mimic using Lipofectamine 3000. Forty-eight hours after transfection, luciferase activity was measured using a dual-luciferase reporter gene assay system (Vazyme, China) according to the reagent instructions.

### 2.17 Immunohistochemistry

After tissue sections were cut to approximately 4 μm thickness, they were first dewaxed and hydrated, followed by antigen retrieval. Tissue sections were first blocked to inhibit endogenous peroxidase activity and reduce nonspecific binding. They were then sequentially incubated with primary and secondary antibodies targeting the target protein. Staining was performed using DAB chromogenic agent, and finally, hematoxylin counterstaining was used to enhance tissue contrast and facilitate microscopic observation.

### 2.18 Statistical analysis

All experimental data are presented as mean ± standard deviation (mean ± SD) and were derived from at least three independent biological replicates. Statistical differences between groups were assessed using Student’s t-test or two-way ANOVA, depending on the experimental design. Survival outcomes were analyzed using the Kaplan-Meier method, and differences between groups were compared using the log-rank test. A p-value < 0.05 was considered statistically significant. Statistical processing and graphing were performed using GraphPad Prism 9.0 and R software (version 4.2.2), respectively.

## 3 Results

### 3.1 Identification of the key glioma gene SIX5 based on multi-dataset integration and machine learning strategies.

**Figure 1:**
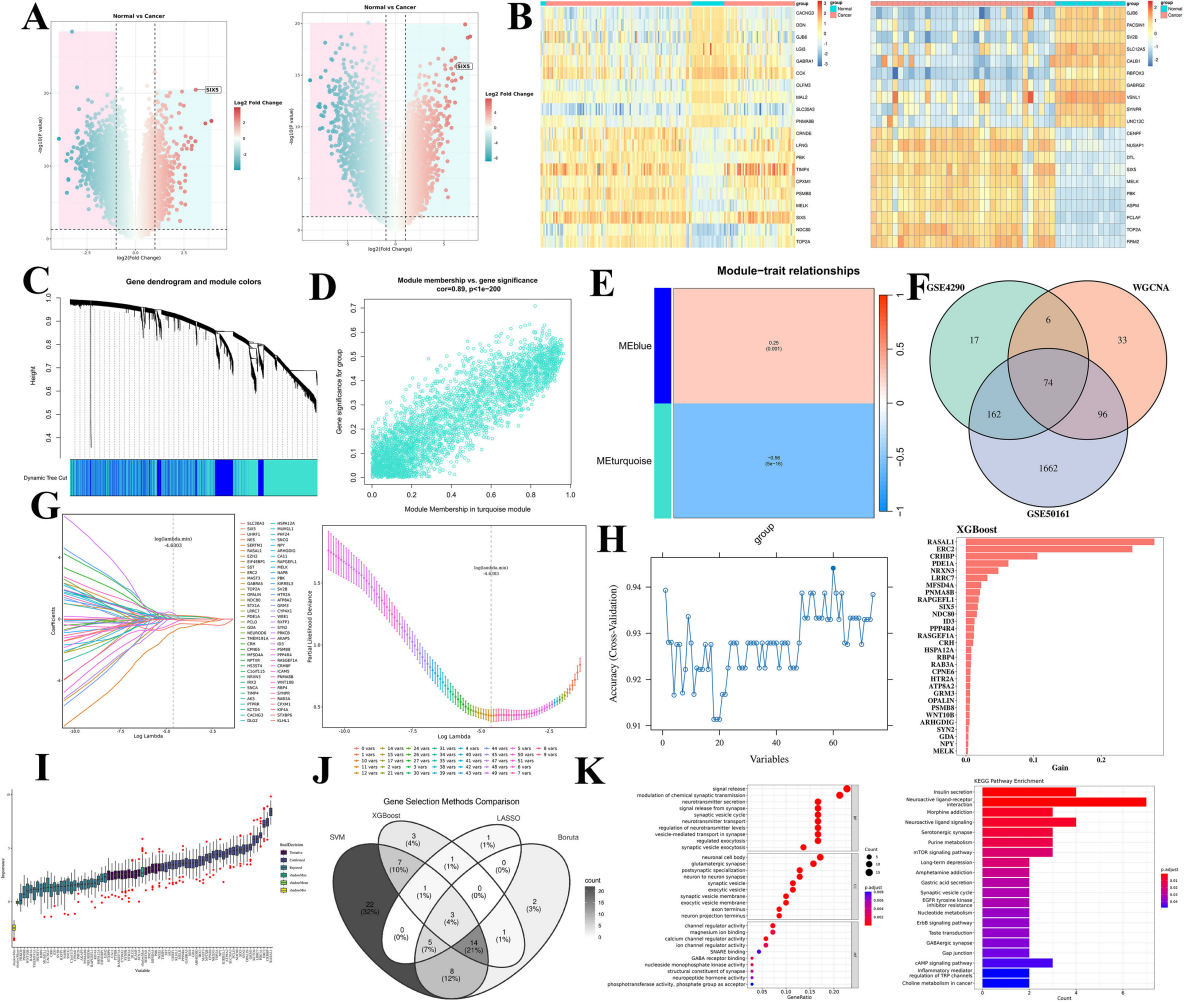
Bioinformatic workflow for identifying key glioma genes through integrated multi-dataset and machine-learning strategies. **(A)** Volcano plots of differentially expressed genes (DEGs). Display the DEGs between glioma and normal tissues from the GSE4290 and GSE50161 datasets. Dashed lines indicate the significance thresholds (|log2FC| > 1, P < 0.05). Red and blue dots represent significantly upregulated and downregulated genes, respectively. **(B)** Heatmap of DEGs and Module-Trait Relationships. Left: Expression patterns of DEGs across glioma and normal samples (red: high expression; blue: low expression). Right: Heatmap showing the correlation between WGCNA modules and clinical traits. **(C)** Clustering dendrogram of Weighted Gene Co-expression Network Analysis (WGCNA). Shows hierarchical clustering of genes into distinct co-expression modules, with each module represented by a unique color (Dynamic Tree Cut). **(D)** Scatter plot of Module Membership versus Gene Significance for the turquoise module. Illustrates the correlation strength between genes within the turquoise module (MEturquoise) and the glioma phenotype, indicating a significant negative correlation. **(E)** Heatmap of Module-Trait Relationships. Quantitatively presents the correlation coefficients and significance levels between each co-expression module and the glioma phenotype, highlighting a positive correlation for the blue module (MEblue) and a negative correlation for the turquoise module (MEturquoise). **(F)** Venn diagram of candidate gene set intersection. Depicts the overlap among DEGs from GSE4290, DEGs from GSE50161, and core genes from WGCNA modules, yielding 74 high-confidence candidate genes. **(G)** LASSO regression analysis. Left: Coefficient trajectories of genes plotted against the log Lambda parameter. Right: Cross-validation deviance curve; the dotted vertical line indicates the optimal Lambda value selected for the model. **(H)** Model predictive accuracy validation curve. Shows the cross-validated accuracy of the LASSO model across different numbers of retained features. **(I)** Feature importance ranking by XGBoost. Ranks candidate genes based on their predictive contribution (importance score), with top-ranking genes labeled. **(J)** Venn diagram for intersection analysis of four machine-learning algorithms. Illustrates the overlap of feature selection results from LASSO, Random Forest, Support Vector Machine (SVM), and XGBoost. The central intersecting region represents the core predictive genes identified by all algorithms, including SIX5. **(K)** Functional enrichment analysis of core genes. Left: Bubble chart of enriched Gene Ontology (GO) biological processes (bubble size corresponds to gene count; color represents enrichment significance). Right: Bar chart of significantly enriched Kyoto Encyclopedia of Genes and Genomes (KEGG) signaling pathways for the core gene set.

To systematically identify key molecular markers for glioma development and progression, this study integrated and analyzed differential expression from two independent gene expression datasets, GSE4290 and GSE50161. Volcano plots revealed significant differences in gene expression between glioma and normal brain tissue, with SIX5 being significantly upregulated in gliomas (**Figure 1A**). Subsequently, weighted gene co-expression network analysis (WGCNA) was used to construct glioma-related co-expression modules (**Figure 1C**). Correlation analysis between modules and phenotypes showed a significant positive correlation between the MEblue module and the glioma phenotype (r > 0.5, **Figure 1D**), while the MEturquoise module showed a significant negative correlation (r < -0.5, **Figure 1E**). Further intersection analysis of the differentially expressed genes from the two datasets with the WGCNA core module yielded 74 high-confidence candidate genes (**Figure 1F**).

To screen core predictive genes, this study employed four machine learning methods: LASSO regression, random forest, support vector machine recursive feature elimination (SVM-RFE), and XGBoost for feature selection. The Lambda optimization curve and cross-validation of LASSO regression showed good predictive performance; XGBoost feature importance ranking further identified key genes such as RNASEL, CRABP2, and CXCL13. Based on the cross-Venezuel analysis results of the four algorithms, SIX5 simultaneously met the conditions of significant differential expression, core localization in the co-expression network, and consistency in screening across multiple algorithms, and was therefore identified as a core predictive gene for glioma (**Figures 1G–J**).

Functional enrichment analysis (**Figure 1K**) showed that representative genes centered around SIX5 are mainly involved in biological processes such as immune regulation, extracellular matrix remodeling, and angiogenesis, and are enriched in glioma-related pathways such as chemokine signaling, cell adhesion, and antigen processing and presentation, providing a theoretical basis for further elucidating the role of SIX5 in glioma progression.

### 3.2 SIX5 is highly expressed in GBM and enriched in specific cellular subpopulations

**Figure 2:**
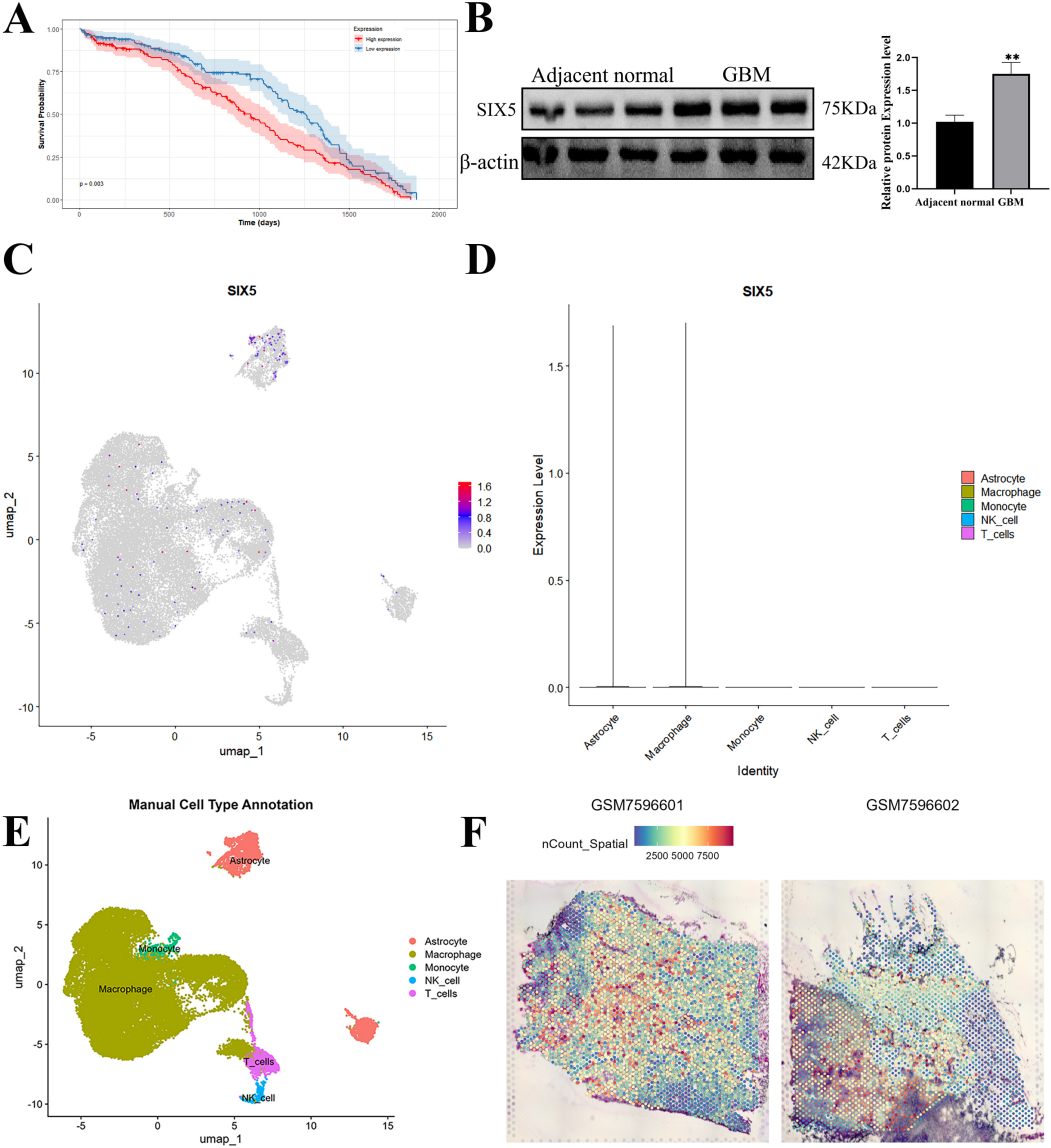
High Expression of SIX5 in Glioblastoma and Its Prognostic Significance**. (A)** Kaplan-Meier survival curve analysis based on the TCGA database showed that the overall survival of patients in the high SIX5 expression group was significantly shorter than that in the low expression group (p = 0.003). **(B)** SIX5 protein levels in paired adjacent normal brain tissue and glioblastoma tissue were detected by Western blot, with β-actin as an internal control; the right-hand bar chart shows the quantitative results of relative protein expression (mean ± standard deviation, p < 0.01). **(C)** Single-cell RNA sequencing data were visualized using UMAP dimensionality reduction to show the distribution of SIX5 in various cell subpopulations. **(D)** Bar chart of SIX5 expression levels in different cell types (astrocytes, macrophages, monocytes, NK cells, and T cells). **(E)** UMAP atlas labeled with cell types; different colors represent different cell populations. **(F)** Spatial distribution map of SIX5 expression based on spatial transcriptome samples GSM7596602 and GSM7596601. The color gradient from blue to red indicates that the expression level is from low to high.

Comprehensive analysis of the TCGA and UALCAN databases revealed significantly higher SIX5 expression in glioblastoma (GBM) tissues compared to normal brain tissues. Patients were divided into high-expression and low-expression groups based on the median SIX5 expression, and Kaplan-Meier survival analysis was performed. The results showed that the overall survival of patients in the high-expression group was significantly shorter (P < 0.05) (**Figure 2A**). This finding was further validated in our clinical samples: Western blot analysis showed significantly higher SIX5 protein levels in GBM tissues compared to adjacent normal tissues (**Figure 2B**).

Further single-cell RNA-seq and spatial transcriptome analysis revealed high SIX5 expression in the UMAP dimensionality reduction plot, along with significant cellular heterogeneity. The gene was primarily enriched in astrocyte-like and tumor stem cell-like subsets, while expression levels were lower in endothelial cells and microglia (**Figures 2C, D**). Spatial transcriptional mapping further revealed that SIX5 was distributed in a gradient in tumor tissue, with high expression regions mainly concentrated in the tumor core and invasion front, and often adjacent to necrotic areas. This suggests that SIX5 may play an important role in the progression and invasion of GBM (**Figures 2E, F**).

### 3.3 SIX5 knockdown suppresses malignant phenotypes of GBM cells and attenuates EMT and PI3K/Akt signaling

**Figure 3:**
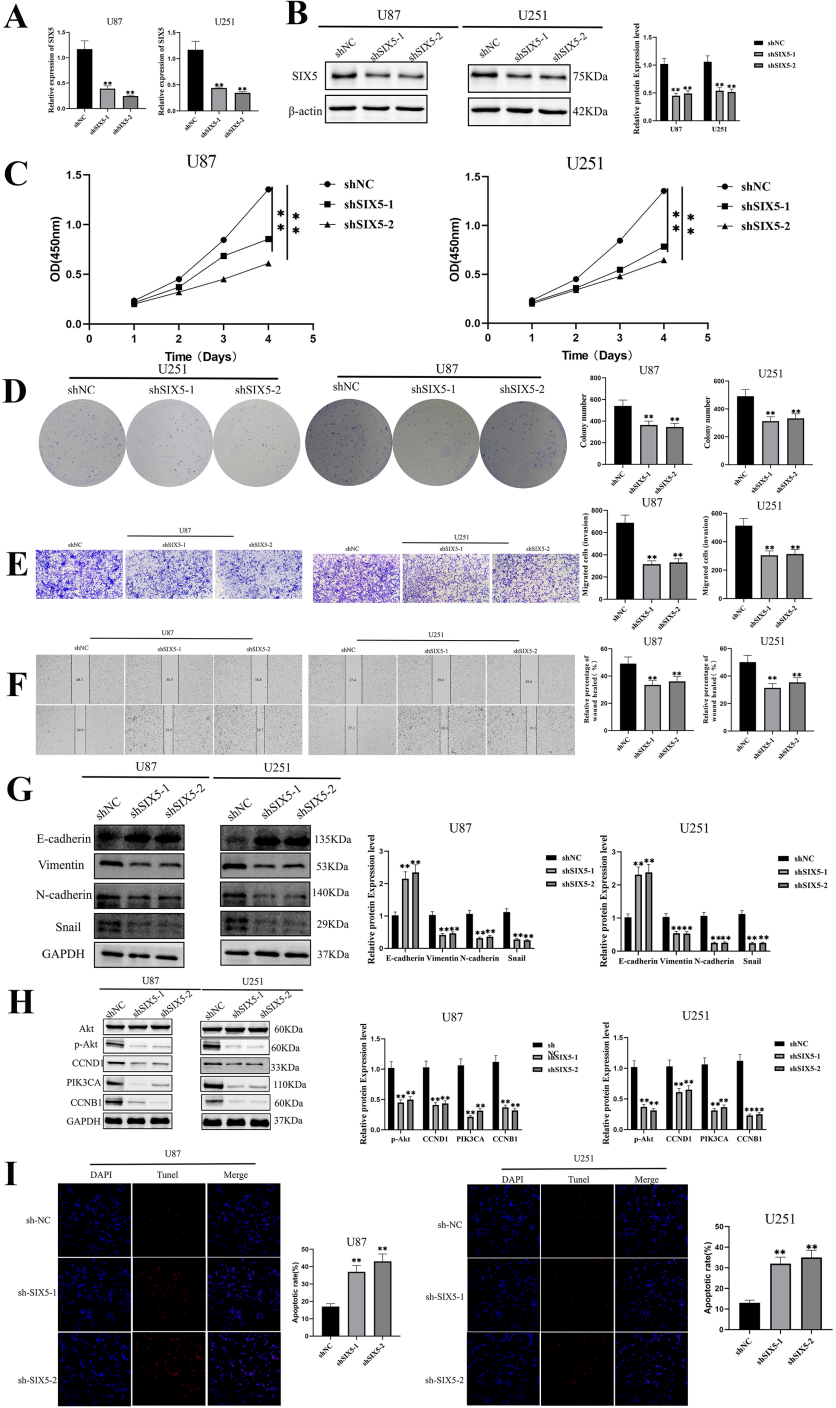
SIX5 Knockdown Suppresses Malignant Phenotypes of GBM Cells and Inhibits EMT and PI3K/Akt Signaling**. (A)** qRT-PCR was used to detect the relative expression levels of SIX5 mRNA in U87 and U251 cells (mean ± standard deviation, p < 0.01). **(B)** Western blot analysis was performed on SIX5 protein expression in the shNC control group and two independent SIX5 knockdown groups (shSIX5-1, shSIX5-2), using β-actin/GAPDH as an internal control; the right-hand graph shows the quantitative analysis results (p < 0.01). **(C)** CCK-8 assays showed that SIX5 knockdown significantly inhibited the proliferation of U87 and U251 cells within 0–4 days (p < 0.01, *p < 0.001). **(D)** Clonogenesis assays showed that SIX5 knockdown significantly reduced the number of cell clones (p < 0.01). **(E)** Transwell migration assays showed that SIX5 knockdown significantly inhibited cell migration (crystal violet staining, p < 0.01). **(F)** Transwell invasion assays showed that SIX5 knockdown significantly reduced cell invasion (p < 0.01). **(G)** Western blot analysis of EMT-related proteins (E-cadherin, Vimentin, N-cadherin, Snail) expression in SIX5 knockdown cells; the right-hand graph shows the quantitative analysis results (p < 0.01, *p < 0.001). **(H)** Western blot analysis of PI3K/Akt pathway-related proteins (Akt, p-Akt, CCND1, p-GSK3β, CCNB1) expression in SIX5 knockdown cells, using GAPDH as an internal control; the right-hand graph shows the quantitative analysis (*p < 0.05, p < 0.01, *p < 0.001). **(I)** TUNEL assay was used to detect apoptosis in U87 and U251 cells; green TUNEL positive cells indicate apoptosis, and blue DAPI marks the cell nucleus; the chart on the right shows the proportion of apoptotic cells (p < 0.01).

To investigate the biological function of SIX5 in glioblastoma, we established stable SIX5 knockdown cell lines in U87 and U251 cells. GFP fluorescence detection showed that the infection efficiency of both lentiviral vectors (shCtrl and shSIX5) exceeded 80%, indicating successful transfection. RT-qPCR analysis showed that the SIX5 mRNA level in the shSIX5 group was significantly decreased compared to the shCtrl group (**Figure 3A**). Further Western blot results showed that SIX5 protein expression was significantly reduced in the knockdown cells (**Figure 3B**), validating the effectiveness of gene knockdown.

Functional experiments showed that SIX5 knockdown significantly altered GBM cell behavior. CCK-8 assay results showed that the OD450 values of U87 and U251 cells in the shSIX5 group were significantly lower than those in the control group, suggesting suppressed cell proliferation (**Figure 3C**). Clonogenesis assays also showed that SIX5 deficiency led to a significant reduction in clone number (**Figure 3D**). Scratch assays showed that shSIX5 cells migrated at a significantly lower rate than the control group, while Transwell assays further confirmed a significant decrease in their invasive ability (**Figures 3E, F**), indicating that SIX5 knockdown effectively inhibits the migration and invasion of GBM cells.

Considering the role of epithelial-mesenchymal transition (EMT) in tumor invasion, we examined the expression of EMT-related markers N-cadherin, Snail, and Vimentin. Results showed that SIX5 knockdown significantly reduced the expression levels of these EMT-related marker proteins (**Figure 3G**). Furthermore, the PI3K/Akt signaling pathway and key proteins regulating the cell cycle were also affected: phosphorylated Akt (p-Akt), PIK3CA, CCND1, and CCNB1 proteins were all decreased in shSIX5 cells (**Figure 3H**). TUNEL staining results showed that the apoptosis rates of U87 and U251 cells in the shSIX5 group were significantly higher than those in the shCtrl group, indicating that SIX5 deficiency promotes GBM cell apoptosis (**Figure 3I**). In conclusion, SIX5 knockdown significantly inhibited the proliferation, colony formation efficiency, and migration and invasion potential of GBM cells, while simultaneously enhancing apoptosis. Correspondingly, the protein expression levels of EMT markers and key molecules in the PI3K/Akt pathway were downregulated, suggesting that SIX5 may promote the malignant phenotype of GBM cells by regulating related signaling pathways.

### 3.4 KDM5C regulates transcriptional activation of SIX5

**Figure 4:**
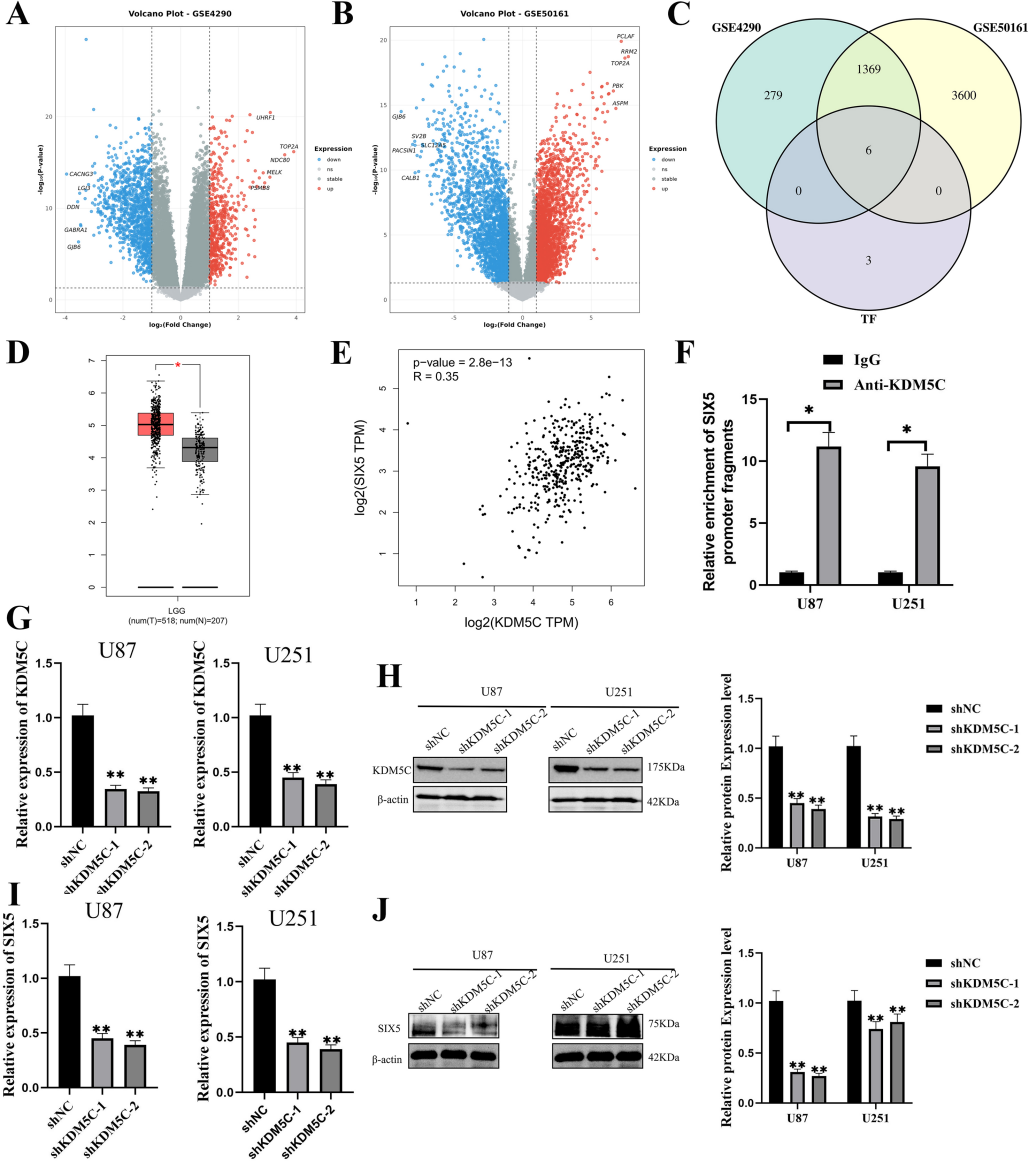
KDM5C Positively Regulates SIX5 Expression. **(A, B)** Volcano plots show differentially expressed genes between glioma and normal brain tissue in the GSE4290 **(A)** and GSE50161 **(B)** datasets, with red indicating upregulation, blue indicating downregulation, and gray indicating no significant difference; dashed lines mark the significance threshold. **(C)** Venn plots show the intersection of GSE4290, GSE50161, and the transcription factor prediction library, identifying 6 shared genes. **(D)** Box plots show the differential expression of SIX5 in low-grade gliomas (LGG, n=518) and glioblastomas (GBM, n=207). **(E)** Scatter plots show a significant positive correlation between SIX5 and KDM5C expression (p = 2.8e-13, R = 0.35). **(F)** ChIP results showed that the KDM5C antibody significantly enriched the SIX5 promoter fragment in U87 and U251 cells, with IgG serving as a negative control (p < 0.05). **(G, H)** qRT-PCR and Western blot analyses indicated that KDM5C knockdown significantly reduced the mRNA and protein levels of this gene in U87 and U251 cells (mean ± standard deviation, p < 0.01). **(I, J)** Further qRT-PCR and Western blot analysis confirmed that KDM5C knockdown significantly downregulated SIX5 mRNA and protein expression (p < 0.01). All experiments were performed at least three times independently. Data are presented as mean ± SD. Statistical analyses were performed using Student’s t-test or one-way ANOVA; *p < 0.05, **p < 0.01 denote statistical significance.

To investigate the upstream transcriptional regulatory mechanisms underlying SIX5 overexpression in glioblastoma (GBM), we first utilized the hTFtarget database to predict candidate transcription factors potentially regulating SIX5 transcriptional activity. Subsequently, multiple glioma-related transcriptomic datasets from the NCBI GEO database, including GSE4290 and GSE50161, were subjected to differential expression analysis. By intersecting the differentially expressed transcription factors with the hTFtarget predictions, six potential key regulators of SIX5 were identified (**Figures 4A–C**). Notably, KDM5C was predicted as a central regulatory gene and biomarker in glioma and exhibited a significant upregulation trend across multiple GBM transcriptomic datasets. Further validation using the UALCAN database on the TCGA-GBM cohort revealed that KDM5C expression was significantly higher in GBM tumor tissues compared with normal brain tissues (**Figure 4D**), and its mRNA expression was positively correlated with SIX5 expression (**Figure 4E**). Chromatin immunoprecipitation (ChIP) assays demonstrated that KDM5C was highly enriched at the SIX5 promoter region (**Figure 4F**). To further clarify the biological function of KDM5C in GBM and its regulatory relationship with SIX5, stable KDM5C knockdown models were established in U87 and U251 glioma cell lines (**Figures 4G, H**). RT-qPCR and Western blot analyses showed that KDM5C depletion significantly reduced both mRNA and protein expression levels of SIX5 compared with the shCtrl group (**Figures 4I, J**). Collectively, these results indicate that KDM5C directly binds to the SIX5 promoter to positively regulate its transcription, playing a critical regulatory role in glioblastoma.

### 3.5 SIX5 downregulates UBE2C expression to activate the AKT/mTOR pathway and mediate the Warburg effect

**Figure 5:**
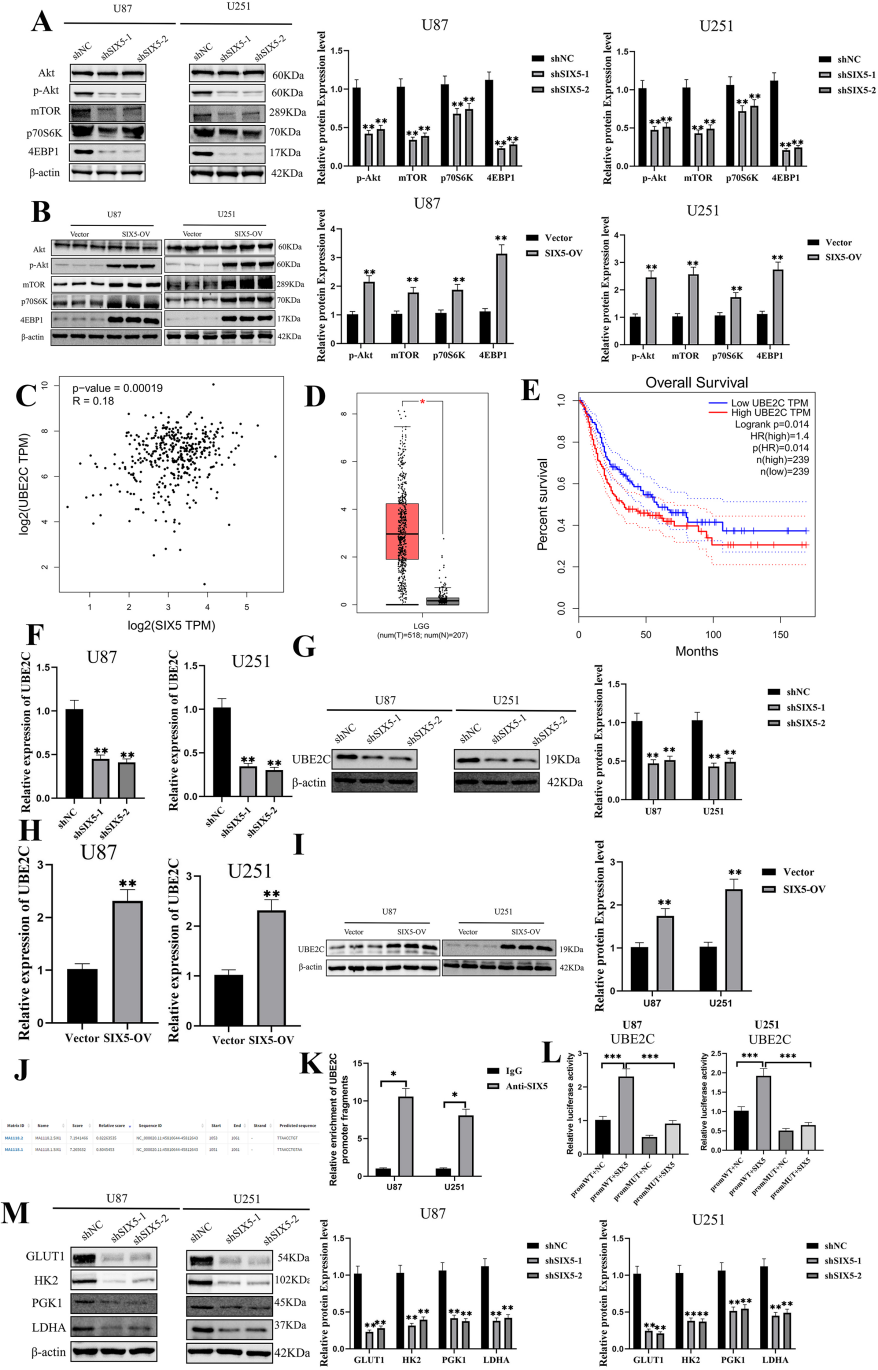
SIX5 Regulates UBE2C Expression and Activates AKT/mTOR Signaling to Mediate the Warburg Effect. **(A, B)** Western blot analysis of key mTOR pathway proteins (Akt, p-Akt, mTOR, p70S6K, 4EBP1) in U87 and U251 cells following SIX5 knockdown **(A)** or overexpression **(B)**, with β-actin as the loading control; right panels show quantitative analysis of relative protein levels (mean ± SD, **p < 0.01, ***p < 0.001). **(C)** Correlation analysis based on the TCGA database showing a significant positive association between SIX5 and UBE2C expression (p = 0.00019, R = 0. 18). **(D)** Boxplot illustrating differential expression of UBE2C in LGG (n = 518) and GBM (n = 207). **(E)** Kaplan–Meier survival analysis indicating significantly reduced overall survival in patients with high UBE2C expression (Log-rank p = 0.014); hazard ratio analysis shows high UBE2C expression is associated with poor prognosis (HR = 1.4, p = 0.014). **(F, G)** qRT-PCR and Western blot analyses showing that SIX5 knockdown significantly decreases UBE2C mRNA and protein levels (**p < 0.01). **(H, I)** qRT-PCR and Western blot analyses showing that SIX5 overexpression significantly upregulates UBE2C mRNA and protein levels (**p < 0.01). **(J)** Bioinformatic prediction of potential SIX5 binding sites in the UBE2C promoter, including location, sequence, and score. **(K)** ChIP assay validating direct binding of SIX5 to the UBE2C promoter in U87 and U251 cells, with IgG as negative control (*p < 0.05). **(L)** Dual-luciferase reporter gene assays in U87 and U251 cells showed that SIX5 significantly enhanced the transcriptional activity of the wild-type UBE2C promoter (***p < 0.001). **(M)** Western blot analysis of the expression levels of key Warburg pathway–associated proteins (GLUT1, HK2, PGK1, and LDHA) in U87 and U251 cells following SIX5 knockdown, with β-actin used as the loading control; the right panel shows quantitative analysis of relative protein expression (mean ± SD, **p < 0.01, ***p < 0.001). All experiments were independently repeated at least three times. Data are presented as mean ± SD. Statistical analyses were performed using Student’s t-test or one-way ANOVA; *p < 0.05, **p < 0.01, ***p < 0.001 denote statistical significance.

To explore the downstream mechanisms by which SIX5 promotes the malignant phenotype of GBM, Western blot analysis was performed. SIX5 knockdown significantly reduced the phosphorylation of AKT and mTOR, as well as downstream effectors p70S6K and 4EBP1, whereas SIX5 overexpression produced the opposite effect (**Figures 5A, B**). Considering how SIX5 activates the AKT/mTOR signaling pathway, we hypothesized that, as a transcription factor, SIX5 may transcriptionally activate key target genes. Analysis of the GEPIA database revealed that UBE2C exhibited the strongest co-expression with SIX5 in GBM samples, attracting our attention. Additional datasets from GEO also showed a strong correlation between UBE2C and SIX5 expression (**Figure 5C**).

Notably, UBE2C is highly expressed in tumors, and its elevated expression is associated with poorer overall survival (OS) and higher pathological stage (**Figures 5D, E**). SIX5 knockdown led to downregulation of UBE2C at both mRNA and protein levels (**Figures 5F, G**), whereas SIX5 overexpression resulted in upregulation of UBE2C (**Figures 5H, I**), strongly suggesting that SIX5 acts as a transcriptional regulator of UBE2C.

Due to the lack of high-confidence SIX5 binding motifs in the JASPAR core database (potentially reflecting limited experimental data on direct binding), and considering the high conservation of homeomorphic domains among SIX family members, this study used the SIX1 binding motif to predict potential SIX5 binding sites in the UBE2C promoter. The prediction results showed multiple potential SIX5 binding sites in the UBE2C promoter (**Figure 5J**). Chromatin immunoprecipitation (ChIP) experiments further validated that SIX5 can directly bind to specific sites in the UBE2C promoter (**Figure 5K**). To further confirm this binding function, luciferase reporter gene assays were performed. The results showed that, compared to the wild-type promoter, luciferase activity was significantly decreased after mutation at the predicted binding site; however, the binding of SIX5 to the wild-type UBE2C promoter significantly enhanced the luciferase signal (**Figure 5L**), suggesting that SIX5 can regulate the transcriptional activity of UBE2C by directly binding to the promoter region. Moreover, Western blot analysis of key glycolytic enzymes, including GLUT1, HK2, PGK1, and LDHA, demonstrated that SIX5 knockdown significantly decreased their protein expression compared with sh-NC controls (**Figure 5M**). Collectively, these results demonstrate that SIX5 functions as a transcriptional activator of UBE2C and mediates the Warburg effect in GBM.

### 3.6 UBE2C is a critical mediator of six5-induced AKT/mTOR signaling activation and malignant phenotypes in GBM

**Figure 6:**
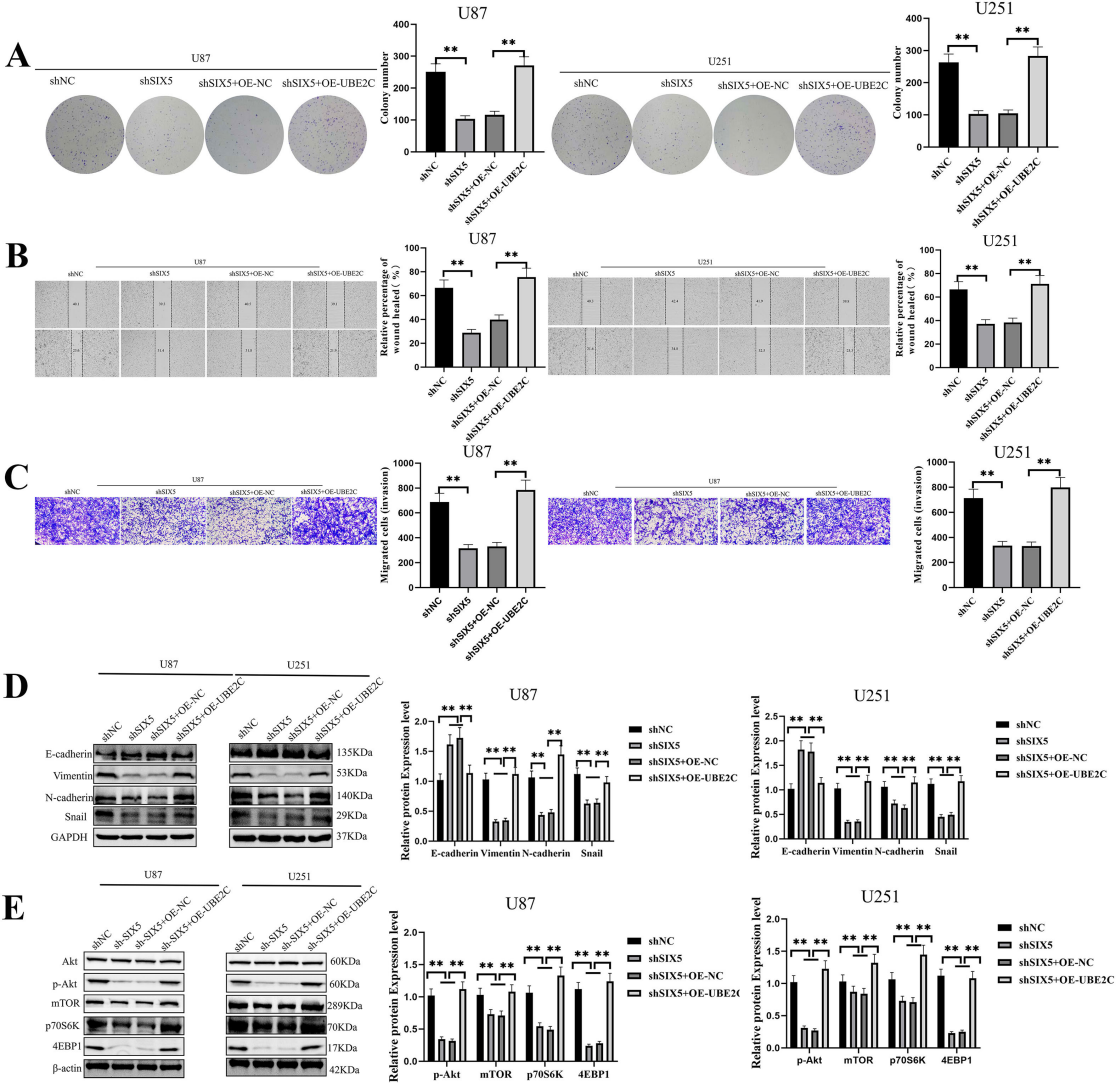
UBE2C Is a Critical Mediator of SIX5-Induced AKT/mTOR Signaling Activation and Malignant Phenotypes in GBM**. (A)** Colony formation assays showed that SIX5 knockdown significantly inhibited the colony formation ability of U87 and U251 cells, while UBE2C overexpression partially reversed this inhibition (p < 0.01). **(B)** Cell scratch assays showed that UBE2C overexpression alleviated the decreased migration ability caused by SIX5 knockdown (p < 0.01). **(C)** Transwell migration assays further verified that UBE2C overexpression enhanced the migration ability of SIX5 knockdown cells (p < 0.01). **(D)** Western blot analysis of EMT-related marker proteins (E-cadherin, Vimentin, N-cadherin, Snail) showed that UBE2C overexpression partially restored the inhibitory effect of SIX5 knockdown on the EMT process (p < 0.01, p < 0.001). **(E)** Western blot analysis of key proteins in the mTOR pathway (Akt, p-Akt, mTOR, p70S6K, 4EBP1) showed that UBE2C overexpression could reactivate the mTOR signaling pathway that was suppressed by SIX5 knockdown (p < 0.01).All experiments were independently repeated at least three times. Data are expressed as mean ± standard deviation. Statistical analysis was performed using Student’s t-test or one-way ANOVA; **p < 0.05, **p < 0.01 indicated statistical significance. OE, Overexpression group; NC, Negative control group.

Although the aforementioned studies confirmed the regulatory role of SIX5 in UBE2C expression, it remains unclear whether SIX5 promotes tumor progression by activating the AKT/mTOR signaling pathway through UBE2C. Therefore, we transfected SIX5-knockdown U87 and U251 cells with a UBE2C overexpression plasmid. Clonogenesis assays showed that UBE2C overexpression significantly enhanced the proliferation of both cell lines and partially reversed the proliferation inhibition caused by SIX5 deficiency (**Figure 6A**). Scratch assays further demonstrated that UBE2C upregulation significantly increased cell migration speed, counteracting the inhibitory effect of SIX5 knockdown (**Figure 6B**). Transwell invasion assays also showed that UBE2C overexpression significantly enhanced cell invasion and alleviated the inhibitory effect of SIX5 deficiency (**Figure 6C**). Furthermore, UBE2C overexpression was able to rescue SIX5 knockdown-induced G2/M phase cell cycle arrest. Western blot analysis of EMT-related biomarkers revealed that upregulation of UBE2C upregulated E-cadherin while downregulating N-cadherin, Vimentin, and Snail, partially reversing the inhibitory effect of SIX5 deficiency on EMT (**Figure 6D**). These results suggest that UBE2C is a key downstream effector molecule in SIX5 activation of the AKT/mTOR signaling pathway and maintenance of the malignant phenotype of GBM cells (**Figure 6E**). In summary, UBE2C plays a central role in SIX5-driven AKT/mTOR signaling pathway activation and GBM malignant progression.

### 3.7 UBE2C reverses the inhibitory effects of SIX5 on GBM cells *in vivo*

**Figure 7:**
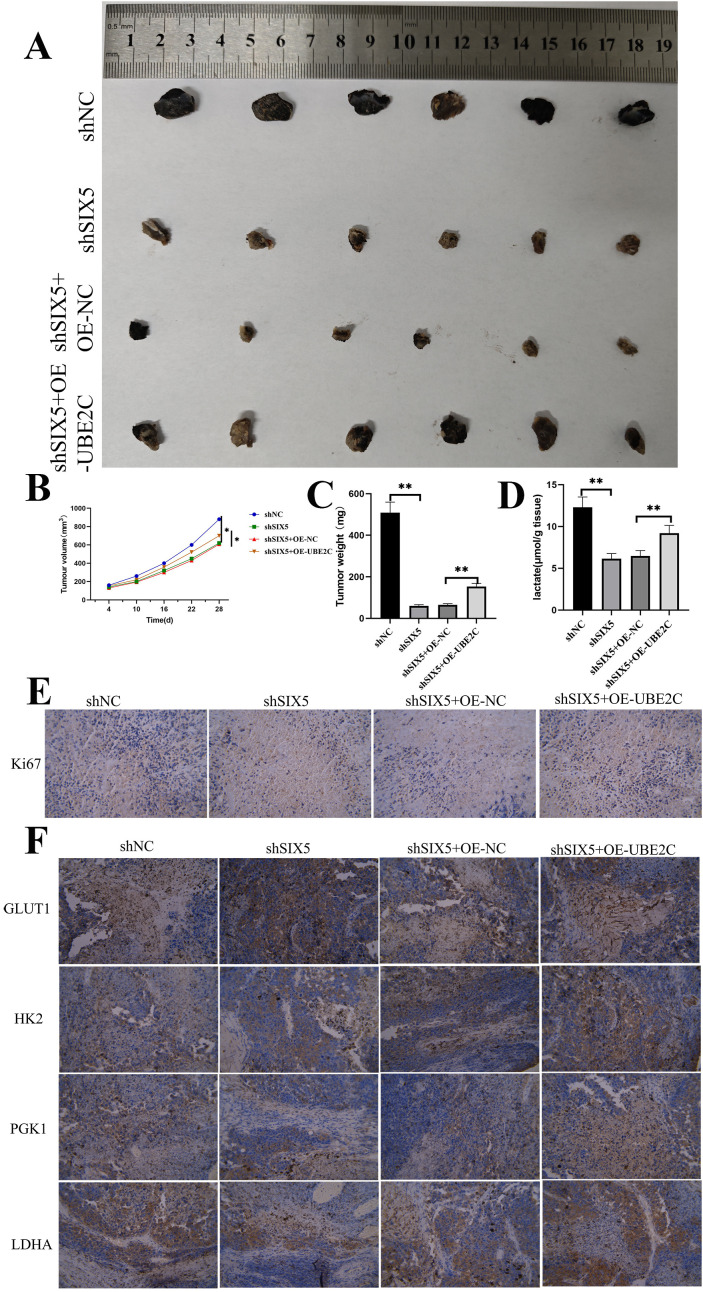
UBE2C reverses the inhibitory effects of SIX5 on GBM cells *in vivo*. **(A)** Representative gross specimens of subcutaneous xenografts in nude mice on day 28. The experiment was divided into four groups: shNC (negative control), shSIX5 (SIX5 knockdown), shSIX5+OE-NC (SIX5 knockdown + empty vector), and shSIX5+OE-UBE2C (SIX5 knockdown + UBE2C overexpression). **(B)** Tumor growth curves showed that SIX5 knockdown significantly inhibited tumor proliferation (P < 0.05), while UBE2C overexpression partially restored its growth capacity. **(C)** Quantitative results of tumor weight on day 28 showed that the tumor size was significantly reduced in the shSIX5 group, while it was significantly increased in the UBE2C overexpression group (P < 0.01). **(D)** Lactate assays in the xenograft tissue showed that SIX5 knockdown reduced lactate production, while UBE2C overexpression partially restored lactate levels (P < 0.01). **(E)** Ki-67 immunohistochemical results showed that the cell proliferation rate decreased in the shSIX5 group, and UBE2C overexpression partially restored proliferative activity. **(F)** Immunohistochemical analysis of glycolysis-related proteins (GLUT1, HK2, PGK1, LDHA) showed that SIX5 knockdown significantly downregulated their expression, while the expression levels were restored in the shSIX5+OE-UBE2C group, suggesting that UBE2C can reverse the glycolysis inhibition caused by SIX5 deficiency. Scale bar: 100 μm. Data are expressed as mean ± standard error, with 6 mice per group. **P < 0.01.

To investigate the role of the SIX5/UBE2C axis in tumor growth and glycolysis-related metabolic alterations, a BALB/c nude mouse subcutaneous xenograft model was established. Results showed that, consistent with *in vitro* experiments, SIX5 knockdown significantly inhibited subcutaneous tumor growth, manifested as a significant reduction in tumor volume and weight; while SIX5 overexpression significantly promoted tumor progression. In the context of SIX5 deficiency, forced expression of UBE2C partially reversed the inhibitory effect on tumor growth, suggesting that the *in vivo* proliferation capacity of GBM depends to some extent on UBE2C downstream of SIX5 (**Figures 7A–C**).

Immunohistochemical analysis showed that SIX5 knockdown significantly inhibited the expression of glycolysis-related proteins (GLUT1, HK2, PGK1, and LDHA), while UBE2C overexpression partially reversed this inhibitory effect. Ki67 staining further indicated that SIX5 deficiency led to a significant decrease in the proportion of proliferating cells, while UBE2C upregulation significantly restored this proportion, suggesting that UBE2C plays a key role in SIX5-mediated cell proliferation regulation (**Figure 7D**).

Furthermore, lactate content detection revealed that SIX5 knockdown significantly reduced lactate levels, while UBE2C overexpression partially restored lactate levels. This further supports the function of the SIX5/UBE2C axis in regulating GBM glycolytic metabolism (**Figures 7E, F**). In summary, *in vivo* experimental results indicate that SIX5 regulates GBM tumor growth and glycolysis-related metabolic activities through a UBE2C-dependent mechanism, thereby promoting tumor malignant progression.

## 4 Discussion

Recent studies have shown that multiple genes and signaling pathways play crucial roles in glioblastoma. Therefore, elucidating their molecular mechanisms and identifying potential therapeutic targets has become a research focus in this field. This study explored the biological function of the transcription factor SIX5 in glioblastoma. Results showed that SIX5 expression was significantly upregulated in tumor tissues, suggesting a potential role in promoting glioblastoma progression. Further *in vivo* and *in vitro* functional experiments demonstrated that knockdown of SIX5 significantly inhibited the malignant phenotype of glioblastoma cells. Downregulation of SIX5 expression suppressed the proliferation and migration of U87 and U251 cells, weakened epithelial-mesenchymal transition, and significantly increased apoptosis levels.

On the other hand, KDM5C has been identified as an upstream regulatory factor of SIX5 in GBM. KDM5C, also known as JARID1C or SMCX, is a specific H3K4me2/3 demethylase in mammalian cells (21). By removing di- and trimethyl groups from histone H3 lysine 4 (H3K4), it modulates gene transcriptional activity (22). Depending on the methylation context, KDM5 proteins can act either as transcriptional activators or repressors (23). Functional studies have shown that the KDM5 family contributes to cancer stem cell properties, participates in DNA repair, promotes epithelial–mesenchymal transition (EMT), and increases intratumoral heterogeneity (24). Aberrant regulation of KDM5 has been associated with key phenotypic alterations in various cancers (25). KDM5C has been reported to function as a tumor suppressor by modulating enhancer activity in breast, clear cell renal, and cervical cancers (26–28). Conversely, other studies have suggested an oncogenic role of KDM5C in specific tumor settings (29, 30). For example, KDM5C promotes colorectal cancer proliferation via the FBXW7–c-Jun axis and contributes to drug resistance by downregulating ABCC1 (30). It is also transcriptionally regulated by BRD4, thereby facilitating castration-resistant prostate cancer (CRPC) proliferation through PTEN suppression (31, 32). In addition, KDM5C has been identified as a female-biased tumor suppressor in acute myeloid leukemia (AML), where it maintains leukemic cell differentiation by removing H3K4me3 bivalent modifications at immature gene promoters (29). However, the mechanistic relationship between KDM5C and SIX5 in glioblastoma (GBM) remains poorly understood.

Although this study mainly focuses on the pro-cancer role of SIX5 in GBM, given the high conservation of SIX family members in terms of domain composition and DNA binding sequence, whether there is a functional compensation or synergistic co-regulatory mechanism between SIX1 and SIX5 still deserves further systematic investigation. At the structural level, the six members of the SIX family (SIX1–SIX6) are divided into three evolutionarily conserved subfamilies based on the sequence similarity between the SIX domain (SD) and the homeobox DNA binding domain (HD): SIX1/SIX2, SIX3/SIX6 and SIX4/SIX5. All members recognize and bind the MEF3 core consensus sequence (TCAGGTTTC) through the highly conserved HD, thereby endowing members of different subfamilies with potential competitive binding ability on specific cis-regulatory elements (11, 33). In fact, studies have confirmed *in vitro* that SIX1, SIX2, SIX4 and SIX5 can specifically bind to the MEF3 site in the myopoietin promoter, suggesting that the above members have inherent structural redundancy at the target promoter binding level (34). At the functional level, both SIX1 and SIX5 have been shown to play a role in promoting tumor progression in GBM. The former drives GBM cell proliferation and invasion by activating connective tissue growth factor (CTGF) through transcription (20), while this study reveals that SIX5 directly binds to the UBE2C promoter and activates its transcription, thereby continuously activating the AKT/mTOR signaling pathway and reshaping the tumor glycolytic metabolic phenotype. Although the downstream target genes of the two are different, the common high expression of SIX1 and SIX5 in GBM suggests that the two may form a synergistic or compensatory regulatory network in the promoter region of shared target genes. This inference is also indirectly supported by the field of developmental biology - SIX1 and SIX4 have been shown to have extensive functional synergy and mutual compensation relationship in muscle, kidney and gonad development (35). Since the SIX5 binding motif data in the JASPAR database is limited, this study uses the SIX1 binding motif to predict potential binding sites of the UBE2C promoter, suggesting that the two may be compatible in DNA recognition sequences. Therefore, the possibility that SIX1 will mediate partial compensatory transcriptional activation by competitively binding MEF3-like elements when SIX5 is suppressed cannot be ruled out (36). However, it should be noted that there is a fundamental difference between SIX1 and SIX5 in their transcriptional activation mechanisms. SIX1 itself lacks an intrinsic transcriptional activation domain, and its transcriptional activation function is highly dependent on coactivators such as Eyes Absent (EYA) family proteins. SIX5, on the other hand, has an independent C-terminal activation domain and can directly drive the transcription of target genes without relying on coactivators (11, 13). This structural difference theoretically determines that even if the two bind to the same target site, their transcriptional activation efficiency will be fundamentally different due to the expression status of EYA protein in the GBM tumor microenvironment.

The ubiquitin-binding enzyme family (E2s) are highly conserved proteins that play a central role in ubiquitin-dependent protein degradation and are widely involved in key biological processes such as cell cycle regulation, signal transduction, and cell differentiation (37, 38). Previous studies have shown that abnormal expression of UBE2C is closely related to the immune microenvironment of various tumors, including immune cell infiltration and expression of immune checkpoint molecules, suggesting that it may serve as a potential biomarker for assessing the response to immunotherapy (39). For example, Wenjie Li et al. reported that UBE2C expression was significantly increased in bladder cancer and was positively correlated with lymphangiogenesis and lymph node metastasis (40); Ruimin Ma et al. further found that as the malignancy of glioma increases, the expression level of UBE2C gradually increases, and its high expression is closely related to disease progression (41). Based on the above background, this study found that SIX5 can enhance the binding to the UBE2C promoter, thereby upregulating the transcription level of UBE2C and ultimately promoting the malignant phenotype development of glioblastoma.

The Warburg effect, a core feature of tumor metabolism, is regulated by multiple signaling pathways, providing potential targets for clinical intervention in tumors (42, 43). Previous studies have shown that the AMPK/mTOR signaling axis plays a crucial role in the regulation of aerobic glycolysis in various tumors (44, 45). For example, Juexian Xiao et al. reported that TRIM27 enhances aerobic glycolysis by activating the AMPK/mTOR pathway, thereby promoting the invasiveness of glioblastoma (46). Furthermore, in glioblastoma, the mTOR/c-Myc axis and AKT-mediated upregulation of GLUT1 and GLUT3 synergistically promote glycolysis (47–49). It is worth noting that multiple studies have further demonstrated that natural compounds can exert anti-glioma effects by targeting these nodes that are highly correlated with glycolysis and AKT/mTOR signaling, providing new ideas for metabolism-dependent intervention. For example, isocucurbitacin B promotes deanchored apoptosis by downregulating CAV1, inhibits glioma cell proliferation, migration, invasion and EMT, and induces G2/M phase arrest and apoptosis; PDX and mouse *in situ* models further verified its tumor-suppressive effect, with CAV1 as the key target (50). Liu et al. found that isoliquiritigenin can downregulate circ0030018, restore the inhibitory effect of miR-1236 on HER2, and inhibit glioma proliferation and invasion (51). In addition, natural polyphenols such as curcumin, resveratrol and quercetin exert anti-GBM effects by inhibiting pathways such as PI3K/AKT/mTOR, AKT/NF-κB, and Wnt/β-catenin, while regulating cell cycle, apoptosis and metabolism (52–56). These studies have confirmed from different dimensions the core driving role of AKT/mTOR and related signaling pathways in the malignant progression of GBM, and also suggest that natural active ingredients can synergistically intervene with the KDM5C–SIX5–UBE2C axis targeting strategy, providing a theoretical basis for multi-target combination therapy.

This study shows that SIX5 can promote the Warburg effect by activating the AKT/mTOR signaling pathway, thereby driving the malignant progression of glioblastoma. SIX5 can specifically bind to the UBE2C promoter region and enhance its transcriptional activity. Notably, downregulating UBE2C expression can significantly reduce the proliferation, migration and invasion of GBM cells mediated by SIX5. It is known that members of the SIX family can drive tumorigenesis through a variety of mechanisms, including EMT, transcription factor interactions and metabolic reprogramming. For example, SIX1 can accelerate the occurrence and metastasis of thyroid tumors by promoting EMT and activating the TGFβ/Smad2/3 pathway; while SIX2 works synergistically with SOX2 and NANOG in renal cell carcinoma, indicating that it plays a key role in maintaining the characteristics of tumor stem cells (57). In conclusion, the SIX5–UBE2C regulatory axis plays a core role in the occurrence and development of GBM and has potential therapeutic target value.

## Data Availability

The data that supports the findings of this study are available within the article. The original data can be obtained by sending an email to the corresponding author.
