# Supplementary material for: KDM5C-regulated SIX5 promotes glioblastoma progression through transcriptional activation of UBE2C and enhancement of the Warburg effect
Source: Front Immunol. 2026 Mar 20;17:1788510. doi: 10.3389/fimmu.2026.1788510 (PMC13047191; doi:10.3389/fimmu.2026.1788510)
Supplement: Supplementary file 1 [file Table1.docx]

**Supplementary Table S1. RT-qPCR Primer Sequences**

| Gene | Sequence（5’-3’） |
| --- | --- |
| KDM5C | Forward: CAGAGGCTGACTTCAGGAGA |
|  | Reverse: TGGTGAGGTTGTTGGTGTTG |
| SIX5 | Forward: AGGACCTGACCAAGGAGGAC |
|  | Reverse: TCCAGGTTGATGGTGAGGTT |
| UBE2C | Forward: AGCAGCGAGGACTTTGAGAA |
|  | Reverse: TGTGAGCAGCAGTCTTGGTG |
| β-actin | Forward:CACCATTGGCAATGAGCGGTTC |
|  | Reverse:AGGTCTTTGCGGATGTCCACGT |

**Supplementary Table S2. Information on Primary Antibodies**

| Name | Cat. | Dilution ratio | Manufacturer | Country |
| --- | --- | --- | --- | --- |
| rabbit anti‑KDM5C | ab34718 | 1：1000 | Abcam | UK |
| rabbit anti-SIX5 | NBP1‑85009 | 1：1000 | Novus Biologicals | USA |
| rabbit anti-UBE2C | ab12290 | 1：1000 | Abcam | UK |
| rabbit anti-β‑actin | ab6276 | 1：1000 | Abcam | UK |

**Supplementary Table S3. Antibodies Used for ChIP Experiments**

| Name | Cat. | Dilution ratio | Manufacturer | Country |
| --- | --- | --- | --- | --- |
| Anti-SIX5 | #8483S | 1:50 | Cell Signaling Technology | USA |
| Normal Rabbit IgG | #2729S | 1:50 | Novus Biologicals | USA |

**Supplementary Table S4. Primers Used for ChIP Assays**

| Gene | Sequence（5’-3’） |
| --- | --- |
| KDM5C | Forward: CAGGAGGAGGAGGAGGAGGA |
|  | Reverse: TCCCTGCTGCTGCTGCTTCT |
| SIX5 | Forward: GAGGAGGAGGAGGAGGAGGA |
|  | Reverse:TCTGCTGCTGCTGCTGTTCT |
| UBE2C | Forward: AGGCTGAGGAGGAGGAGTGA |
|  | Reverse: TCTGCTGCTGCTGCTGTTCT |
